# Long acting reversible contraceptives utilization and associated factors among women of reproductive age in Arsi Negele town, Southeastern Ethiopia

**DOI:** 10.1186/s40834-020-00109-6

**Published:** 2020-06-01

**Authors:** Desalegn Tsegaw Hibstu, Akalewold Alemayehu

**Affiliations:** 1 Department of Reproductive Health, School of Public Health, College of Medicine and Health Sciences, Hawassa University, Hawassa, Ethiopia; 2 Department of Epidemiology, School of Public Health, College of Medicine and Health Sciences, Hawassa University, Hawassa, Ethiopia

**Keywords:** Arsi Negele town, Long acting and reversible contraceptive, Magnitude, Factors

## Abstract

**Background:**

Ethiopia is the second populous country in Africa with a total fertility rate of 4.6 and contraceptive prevalence of 35%, where implant and intrauterine contraceptive devices account for 8 and 2% respectively. The aim of this study was to determine the magnitude of long acting reversible contraceptives utilization and its associated factors among women of reproductive age in Arsi Negele town, Southeastern Ethiopia.

**Methods:**

Facility-based cross-sectional study was conducted from April 01–May 30, 2017. A total of 361 women using modern contraceptives were selected by a systematic random sampling technique. Pre-tested and interviewer administered structured questionnaire was used to collect quantitative data. Bivariate and multivariate logistic regressions were performed using SPSS version 20.0 software.

**Result:**

The magnitude of long acting reversible contraceptives (LARCs) utilization was 33.5% [95% CI, 28.5–38.8]. Husband with no formal education [AOR = 0.41, CI: 0.16, 0.78] and unemployed women [AOR = 0.35, CI: 0.42, 0.65] were negative predictors while having media exposure [AOR = 7.14, CI: 3.85, 13.25], women who desired only one child [AOR = 3.28, CI; 1.28, 8.39] and husband support [AOR = 7.33, CI: 3.48, 15.43] were positive predictors of LARCs utilization.

**Conclusion:**

The overall utilization of LARCs is 33.5%. Creating employment opportunities, male involvement, advertisement and advocacy activities through mass media need to be considered to improve utilization of LARCs.

## Background

Long acting reversible contraceptives (LARCs) are birth controls providing effective pregnancy protection for an extended duration without user action. These methods include implants and intrauterine contraceptive devices (IUCDs) [1].

Globally in 2017, 12% of married women were estimated to have an unmet need for family planning. The severity was higher in Africa (22%) compared to other regions, where the unmet need for family planning is estimated to be as low as 10 % in developed countries [2].

In the developing world, two to three among ten women using oral contraceptives or injectable discontinued within 2 years of use due to side effects, use inconsistency, user run out of the method or other related concerns and as a result majority of women can be benefited if they use LARCs [3].

According to the 2007 population census, Ethiopia is the second populous country in Africa next to Nigeria [4] with a total fertility rate of 4.6 and contraceptive prevalence of 35%. Of the total contraceptive utilization, long acting and permanent contraceptive methods account for 10%, IUCD and implant account for 2 and 8% respectively [5].

Depending on setting, a number of factors have been described in literature to affect adoption of LARCs among contraceptive users. Evidence suggest from other countries and in Ethiopia indicated that desire to more children, opposition to use, lack of knowledge, side effects of contraceptives, age of women, educational status of women and men, and family income are the potential factors affecting utilization of LARCs [4, 6].

The Federal Ministry of Health, Ethiopia considered the role of LARCs for birth spacing and limiting, and planned to increase implant and IUD to 33 and 15% respectively in the method mix by the end of 2020 [7]. In Ethiopia, all contraceptives including long acing and permanent contraceptive methods are rendered free [8]. In the country, many governmental and nongovernmental organizations are delivering LARCs through static and outreach outlets at all levels of health facilities (hospitals, health centers and health posts) [9].

Increasing contraceptive method mix played a big role in reducing discontinuation rates due to short-acting hormonal methods that are primarily chosen by contraceptive users in SSA [7, 10] where about 1.8 million unplanned pregnancies would been avoided within 5 years if one-fifth of women utilizing oral contraceptives and injectable shift to LARCs [11]. Therefore, the aim of this study was to determine the magnitude of long acting and permanent contraceptive utilization among women of reproductive age using modern contraceptives in Arsi Negele town, southeastern Ethiopia.

## Methods

### Study design and setting

Facility-based quantitative cross-sectional study was employed from April 01–May 30, 2017. The study was conducted in Aresi Negelle town. The town is located in West Arsi Zone of Oromia regional state in southeastern Ethiopia at a distance of 225 Kilometer) KM from Addis Ababa, capital city of Ethiopia. According to the 2007 Ethiopian population and housing census, the total population of the town was 36,699 (17,618 female) [Arsi Negele town Health Office, Annual report, 2017, Unpublished].

The town has one health center providing preventive, promotive, curative and rehabilitation services for the community and other surrounding communities. The source populations were all women of reproductive age group living in Aresi Negelle town and the study populations were all women of reproductive age group (15–49 years old) using modern contraceptives in Arsi Negele town. However, women who were diagnosed by a physician as seriously ill and unable to hear were excluded.

### Sample size determination and sampling procedure

Sample size was determined using single population proportion formula considering the following assumption: 95% confidence level (***z***_***α/2***_^***2***^*)*, 4% margin of error (d) and expected proportion (p) of long acting reversible contraceptive users was 19.5% [12].

$$ \frac{n={z_{\alpha /2}}^2p\ \left(1-p\right)}{d^2}=\frac{(1.96)^2x\  0.195(0.805)}{(0.04)^2}=376 $$

Study participants were selected using a systematic random sampling technique with an interval of two where the interval constant was obtained by dividing the total modern contraceptive users in the health center to the sample size from their family planning registration book.

### Data collection tool and procedure

Pre-tested and interviewer-administered structured questionnaire, first prepared in English and translated to Amharic language was employed to collect quantitative data. Six registered female nurses were participated to collect the data. Training was given to data collectors for 2 days. The training focused on the study objectives, questionnaire and process of data collection. One bachelor degree holder with principal investigator supervised the data collection process and checked filled questionnaire for consistency and completeness.

### Statistical analysis

After data collection, data were edited and cleaned before analysis, each questionnaire was checked for completeness and code was provided. Data were entered into computer using EPI Info version 3.5.4 and the analysis was done using SPSS version 20.0. Frequency, percentage and descriptive summaries were used to describe the study variables. Logistic regression was carried out to identify factors associated with utilization of LARCs. Variables with *p*-value ≤0.2 in the bivariate analysis were transferred for multivariable logistic regression analysis to control the effect of confounders. Adjusted odds ratios (AOR) with 95% confidence interval (CI) and *p*-value of less than 0.05 were considered to have significant association between the outcome and the explanatory variables.

## Results

### Socio demographic and economic characteristics

A total of 361 of reproductive aged women were included in the study. The mean age of the study subjects was 27.4 years (+ 4.9, standard deviation (SD)). Two hundred and twenty seven (62.9%) study subjects were found in the age group 25–34 years. One hundred and eighty eight (52.1%) of the respondents were followers of orthodox religion and 191 (52.9%) were Oromo by ethnicity. Among the total study participants, 342 (94.7%) were currently married and about nine in ten, 338 (93.6%) living in urban area.

One hundred and sixty seven (46.3%) women had acquired only primary education and 159 (44%) of all partners had acquired at least a secondary education. As shown in Table 1, about six in ten, (62.3%) were unemployed and 340 (94.2%) of their husbands were employed. Almost one among four, 94 (26%) mothers had monthly income between 1001 and 2000 Ethiopian birr (≈ 46 USD – 93 USD) (Table 1).

**Table 1:** Socio demographic and economic characteristics of the study participants in Arsi Negele town, Southeastern Ethiopia, 2017

Variables	Category	Frequency(n)	Percentage (%)
**Age in years**	15–24	100	27.7.9
	25–34	227	62.9
	> 35	34	9.4
**Ethnicity**	Oromo	191	52.9
	Amhara	91	25.2
	Guraghe	35	9.7
	Tigre	25	6.9
	Wolayita	19	5.3
**Religion**	Orthodox	188	52.1
	Protestant	94	26.0
	Muslim	79	21.9
	Catholic	11	3.0
**Marital status**	Currently married	342	94.7
	Currently unmarried	19	5.3
**Residence**	Urban	338	93.6
	Rural	23	6.4
**Women education**	No formal education	84	23.3
	Primary education	167	46.3
	Secondary & above	110	30.5
**Husband /partner education**	No formal education	59	16.3
	Primary education	143	39.6
	Secondary & above	159	44.0
**Women occupation**	Unemployed	225	62.3
	Employed	136	37.7
**Husband occupation**	Unemployed	21	5.8
	Employed	340	94.2
**Monthly income (Ethiopian Birr)**	< 1000	64	17.7
	1001–2000	94	26.0
	2001–3000	78	21.6
	> 3000	125	34.6

### Reproductive history of women

As indicated in table two below, of the total women, about nine in ten women 322(89.2%) had history of giving birth and 152 (42.1%) gave more than three live births with 151(41.8%) alive currently during the study period. One hundred fifty four (42.7%) women desired to have two and above children in their reproductive life and 109 (30.2%) wish to have more children within the next 2 years. About three fourth 275 (76.2%) of the desired number of children was decided jointly (by the husband and wife). The average number of parity, currently living children, desired number of children in the reproductive life were 2.4 (+ 1.6 standard deviation (SD)), 2.3 (+ 1.5 SD) and 2.4 (+ 1.3 SD), respectively (Table 2).

**Table 2:** Reproductive history of study participants in Arsi Negele town, Southeastern Ethiopia, 2017

Variables	Category	Frequency (n)	Percentage (%)
**History of giving birth**	Yes	322	89.2
	No	39	10.8
**Number of ever born births**	None	39	10.8
	One	74	20.5
	Two	96	26.6
	Three and above	152	42.1
**Number of alive children**	None	42	11.6
	One	72	19.9
	Two	96	26.6
	Three and above	151	41.8
**Desired number of children**	One	86	23.8
	Two	154	42.7
	Three and above	121	33.5
**Desire to have more children**	Yes	109	30.2
	No	252	69.8
**Decision maker on number of children.**	Woman	23	6.4
	Husband	34	9.4
	Jointly^a^	275	76.2
	God	29	8.0

^a^both woman and husband

### Contraceptive related characteristics

As shown in Table 3, of the total modern contraceptives users, nearly four in five women heard about LARCs. Among the total women, 193 (53.5%) had media exposure about LARCs where 170(88.1%) were exposed to Television (TV). Injectable contraceptive method was the most known type by women, 338 (93.6%) and vasectomy was the least known method, 13 (3.6%). More than two in five, 229 (63.4) women mentioned limiting family size, prevents maternal and child health and child spacing as a benefit of LARCs.

**Table 3:** Contraceptive related characteristics of women of reproductive age groups in Arsi Negele town, Southeastern Ethiopia, 2017

Variables	Category	Frequency (n)	Percentage (%)
**Heard of LARCs**	Yes	295	81.7
	No	66	18.3
**Media exposure to LARCs**	Yes	193	53.5
	No	168	46.5
**Type of media exposed**	Television	170	88.1
	Radio	23	11.9
**Known contraceptive**	Pills	301	83.4
	Injectable	338	93.6
	Implant	282	78.1
	IUCD	243	67.3
	Condom	29	8
	Tuba ligation	46	18
	Vasectomy	13	3.6
**Benefits of LARC**^a^	Limiting family size	27	7.5
	Improves maternal and child health	13	3.6
	Child spacing	23	6.4
	Used for all^a^	229	63.4
	Don’t’ know uses	69	19.1
**Utilization of LARC**	Yes	121	33.5
	No	240	66.5
**Type of LARC used (*n*** **= 121)**	Implant	94	26.0
	IUCD	27	7.5
**Contraceptive discussion among couples**	Yes	287	79.5
	No	69	20.5
**Approval of husband to use contraceptive**	Yes	229	63.4
	No	132	36.6

Used for all^a^: indicate benefits of LARC for limiting family size, improves maternal and child health and child spacing

The overall utilization of LARCs among women using modern contraceptive was 121 (33.5%) [95% CI, 28.5–38.8]. Ninety four (26%) and 27 (7.5%) women practiced implant and intrauterine contraceptive device (IUCD). Almost four among five, 287 (79.5%) women had discussion with their husbands on contraceptive utilization and 229 (63.4%) respondents’ utilization of contraceptive was approved by their husbands (Table 3).

### Factors associated with LARC utilization

As indicated in Table 4, in the multivariate logistic regression analysis; husband education, women occupation, media exposure, desired number of children and approval of partner to use contraceptive were examined to be independent predictors of long acting reversible contraceptive methods.

**Table 4:** Multivariate logistic regression model predicting utilization of LARCs among women in Arsi Negele town, Southeastern Ethiopia, 2017

Variables	Category	Use of LARC		*COR* (95% CI)	AOR* (95% CI)	***P***-value
		***No***	***Yes***			
**Age of women (years)**	15–24	79	21	0.30(0.13, 0.68)	0.44(0.14,1.36)	0.154
	25–34	*143*	84	0.66 (0.32, 1.37)	0.50 (0.19,1.35)	0.171
	> 35	*18*	16	1.00	1.00	
**Husband education**	No formal education	47	12	0.48 (0.24, 0.99)	0.41 (0.16,0.78)	0.006
	Primary education	89	54	1.15 (0.72, 1.84)	0.75 (0.65,0.98)	0.005
	Secondary and above	104	55	1.00	1.00	
**Women occupation**	Unemployed	162	63	0.52 (0.33, 0.82)	0.35 (0.42,0.65)	0.001
	Employed	*78*	*58*	1.00	1.00	
**Heard of LARCs**	Yes	180	115	6.39 (2.67, 15.27)	1.50 (0.53,4.29)	0.450
	No	*60*	*6*	1.00		
**Media exposure to LARCs**	Yes	89	104	10.38 (5.84,18.46)	7.14 (3.85,13.25)	0.002
	No	151	17	1.00	1.00	
**Desire for more children**	Yes	81	28	0.59 (0.36, 0.97)	0.73 (0.38,1.38)	0.329
	No	159	93	1.00	1.00	1.00
**Desired number of children**	One	46	40	4.14 (2.20, 7.80)	3.28 (1.28,8.39)	0.023
	Two	94	60	3.04 (1.72, 5.38)	2.58 (1.16,5.71)	0.020
	Three and above	100	21	1.00	1.00	
**Discussion among couples**	Yes	*81*	*28*	2.55 (1.36, 4.78)	0.493 (0.14,1.69)	0.261
	No	159	93	1.00	1.00	
**Husband Support to use contraceptive**	Yes	118	111	2.55 (1.36,4.78)	7.33 (3.48,15.43)	0.000
	No	122	121	1.00	1.00	

The study demonstrated that the probability LARCs utilization among women whose husbands with no formal education was 59% [AOR = 0.41, 95%CI; 0.16, 0.78] less likely to adapt LARCs compared with husbands attended secondary and above education. The study identified that practicing LARCs decreased by 65% among unemployed women compared with employed women [AOR = 0.35, CI: 0.42, 0.65].

Another important factor was media exposure to LARCs. Women having media exposure to LARCs were seven times more likely to use LARCs liken with women with no media exposure [AOR = 7.14, CI: 3.85, 13.25].

On the other hand, desired number of children and husband approval/support were identified as determinants of LARCs utilization. The odds of using LARCs was nearly three times more likely to be increased among women whose desired number of children was one compared to three and above [AOR = 3.28, CI: 1.28, 8.39]. Women who got support/approval from their husband about contraceptive methods were seven times more likely to practice LARCs [AOR = 7.33, CI: 3.48, 15.43].

## Discussion

In the present study, it was indicated that long acting reversible contraceptives (LARCs) utilization was 33.5%. This finding was nearly in line with a study conducted in Ethiopia: Areka town (29.7%) and Adigrat town (37.3%) [13, 14] and higher than studies done in Ethiopia: Debremarkos (19.5%) and Mekelle (12%) [12, 15]. The increased prevalence noticed in this research might be due to the difference in time and study setting (institutional versus community based) and, improved awareness of women and advocacy of long acting and permanent contraceptives by governmental and non-governmental organization through mass media and health professionals.

The most widely used long acting reversible contraceptive method in this study was implant (26%). This result was similar with other findings in Ethiopia: Arbaminch and west Ethiopia, [16, 17]. Only 7.5% of the study participants practiced intrauterine contraceptive (IUCD) device which is nearly similar with a study conducted in Nigeria (7%) [18], and lower than the findings in Indonesia (52%) [19]. The possible explanation in this study could be women might have misconception about IUCD and its side effects such as interference with sexual intercourse, cancer, delays pregnancy, restriction from working normal activity and invasion of privacy during its insertion and removal [15].

In the current study, it was figured out that utilization of LARCs was higher among women whose husbands completed secondary and above education compared with women who did not attend formal education. This finding is agreed with studies in Ethiopia: Adigrat town and west Ethiopia [14, 17]. This might be explained by the fact that there is no question that better educated family members would have access for information on LARC methods and their increased knowledge on modern contraceptives. It is also thought that increased educational status in particular secondary education and above could have a positive outcome service use and female decision-making power on reproductive health services including family planning.

The study also revealed that utilization of LARC was increased among employed women than unemployed women. This finding was consistent with studies in Ethiopia: Adigrat town and west Ethiopia [14, 17]. This could happen because employed women would have a positive outcome in utilization of LARC by having frequent health institution contact to access the service with payment though it is provided free of charge in Ethiopia.

Significant association was observed concerning media exposure. It was figured out that utilization of LARC was more likely to increase among women had exposure of media. This finding is in line with study conducted in Ethiopia [17]. This could be explained as media has a power in explaining the different methods available, their benefits and where the methods are available and enhances women to utilize the methods.

On the other hand, desired number of children and approval of husband/partner plays significant role on utilization of long acting and permanent contraceptive. Women whose desired number of children only one were more likely to use LARC. Women who had approval from their husband/partner were more likely to use LARC. This finding was congruent with the studies done in Ethiopia: Debremarkos, Arbaminch and Butajira [12, 16, 20].

## Limitations

When interpreting the findings of this study, researchers need to consider the following limitations. First, the cross sectional nature of the data had made impossible to arrive at the causal relation between the different explanatory variables and knowledge of long acting and permanent contraceptives. Second, as the study is based on a sample of all reproductive age group women who are using modern contraceptives, possible selection bias needs to be seen since it did not address the main characteristic of the general population in the study.

## Conclusions

In general, in this study, the magnitude of long acting reversible contraceptive was 33.5%. Husband education, women occupational status, media exposure, desired number of children and approval of husband/partner to use contraceptives were predictors of LARCs among women in Arsi Negele town, Southeastern Ethiopia.

Creating employment opportunities, male involvement, advertisement and advocacy activities through mass media need to be considered to improve utilization of LARCs.

## Data Availability

The data that support the findings of this study will be available from the corresponding author upon reasonable request in the form of statistical package for social sciences (SPSS) spread sheet.

## Abbreviations

AOR: Adjusted odd ratio
CI: Confidence Interval
EDHS: Ethiopian Demographic and Health Survey
IUCD: Intra-uterine device
KM: Kilometer
SD: Standard deviation
LARC: Long acting reversible contraceptive
SSA: Sub Saharan Africa
SPSS: Statistical Package Software for Social Sciences
